# Navigating Complexities: Successful Pericystectomy in a South Asian Female With Hepatic and Peritoneal Hydatid Disease

**DOI:** 10.7759/cureus.65724

**Published:** 2024-07-30

**Authors:** Srinivasa Reddy, Rajesh G Gattani, Harshal Tayade, Pankaj Gharde, Darshana Tote, Nitesh Badwaik, Dheeraj P Surya, Mihir Patil, Chahat Singh

**Affiliations:** 1 Surgery, Jawaharlal Nehru Medical College, Datta Meghe Institute of Higher Education and Research, Wardha, IND; 2 General Surgery, Jawaharlal Nehru Medical College, Datta Meghe Institute of Higher Education and Research, Wardha, IND

**Keywords:** surgical management, peritoneal cyst, liver cyst, echinococcus granulosus, hydatid cyst

## Abstract

Hydatid cyst disease, caused by the larval stage of *Echinococcus granulosus*, is a parasitic infection endemic in many regions, including South Asia. We present a case of a 36-year-old South Asian female with concurrent liver and peritoneal hydatid cysts, emphasizing the diagnostic challenges and management complexities associated with this condition. The patient presented with abdominal pain, nausea, and decreased appetite, and imaging studies revealed characteristic cystic lesions in the liver and peritoneum. Initial medical management with albendazole was followed by surgical excision due to inadequate response to therapy. Postoperative care included prophylactic albendazole to prevent recurrence. This case highlights the importance of a multidisciplinary approach involving medical therapy and surgical intervention tailored to the individual patient's needs and disease presentation.

## Introduction

Hydatid cyst disease, caused by the larval stage of the tapeworm *Echinococcus granulosus*, remains a significant public health concern in endemic regions worldwide, including South Asia. The parasite's life cycle involves canines as definitive hosts and herbivores as intermediate hosts, with humans incidentally becoming infected through ingestion of parasite eggs via contaminated food, water, or direct contact with infected animals [1]. Echinococcosis primarily affects the liver (70%) and lungs (20%), though it can manifest in any organ or tissue, termed as extrahepatic or extrapulmonary hydatid disease [2]. Intra-abdominal involvement, particularly in the peritoneum, is less common but can occur due to rupture or dissemination of hepatic cysts or direct infestation through the gastrointestinal tract [3].

Clinical manifestations of hydatid cyst disease vary widely from asymptomatic cysts discovered incidentally on imaging to symptomatic presentations such as abdominal pain, nausea, vomiting, and obstructive symptoms depending on the cyst's location, size, and complications [4]. Diagnosis relies on a combination of clinical suspicion, imaging studies (e.g., ultrasound, computed tomography), and serological tests (e.g., enzyme-linked immunosorbent assay) to confirm the presence of cystic lesions and identify specific antibodies against *Echinococcus* antigens [5]. Treatment strategies encompass medical therapy with benzimidazoles (e.g., albendazole, mebendazole) to reduce cyst viability and size, coupled with surgical intervention for large, symptomatic, or complicated cysts such as those causing mass effect, rupture, or secondary bacterial infection [6]. Surgical options range from cystectomy or pericystectomy to more conservative approaches like cyst puncture-aspiration-injection-reaspiration (PAIR) in select cases [7]. Given the chronicity and potential for recurrence, postoperative management includes long-term pharmacotherapy with albendazole to prevent cyst recurrence and close monitoring through imaging and serological testing to assess treatment efficacy and detect early relapse [8].

## Case presentation

A 36-year-old South Asian female presented with a six-month history of intermittent abdominal pain localized to the right side. The pain was insidious in onset, gradually worsening, and non-radiating. She also reported nausea and decreased appetite but denied fever, vomiting, trauma, or significant medical history. On physical examination, her abdomen appeared scaphoid with a centrally placed umbilicus. There were no palpable masses, tenderness, guarding, or rigidity noted upon abdominal examination.

**Figure 1:**
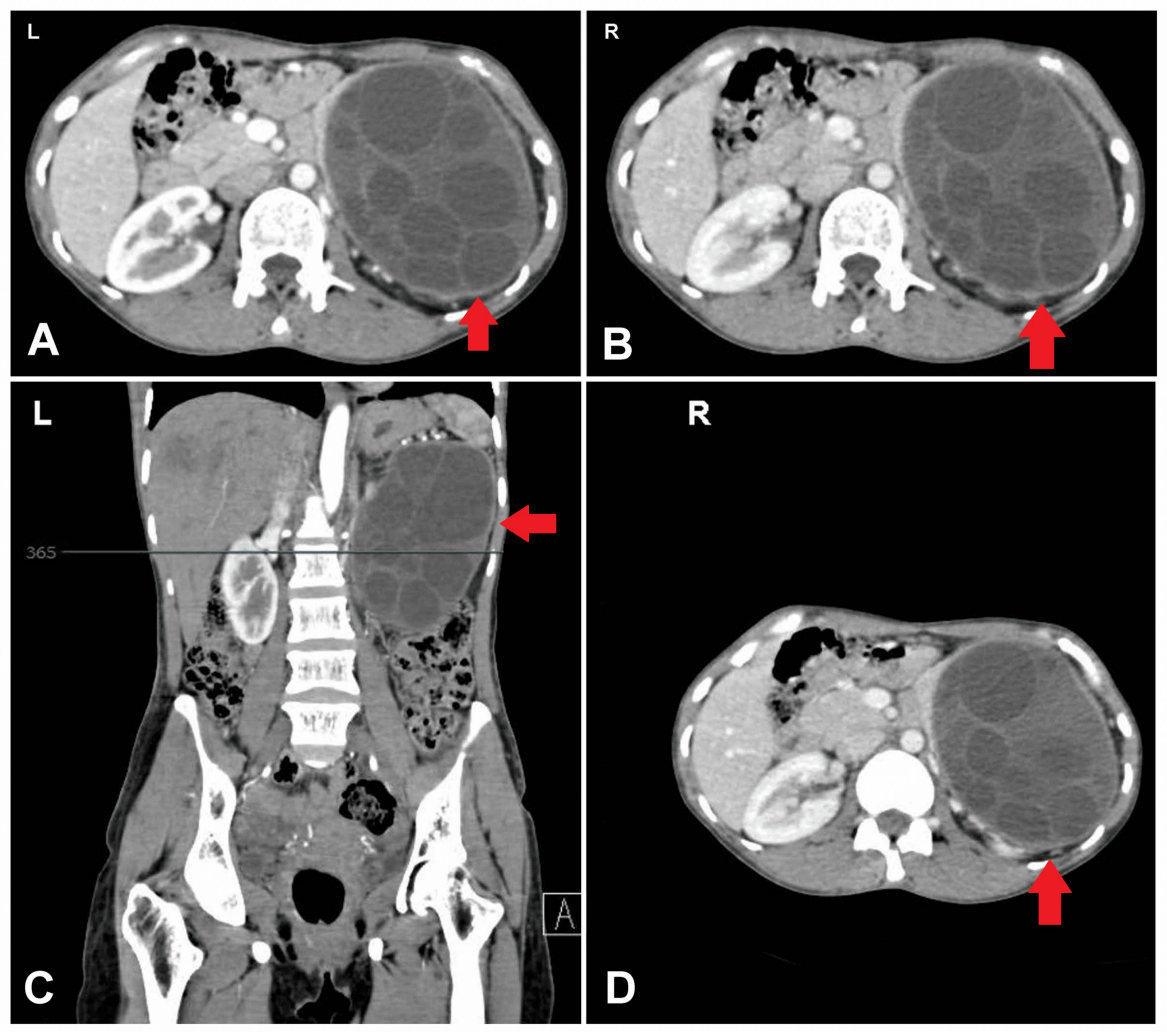
(A-D) CECT abdomen shows cyst measured 9.8 x 10.8 x 13.7 cm and was compressing the left kidney and the tail of the pancreas, closely abutting the splenic and left renal vessels. CECT: Contrast-enhanced computed tomography

Initial investigations including routine blood tests were within normal limits. Contrast-enhanced computed tomography (CECT) of the abdomen and pelvis revealed two significant findings: a well-defined cystic lesion with folded membranes in segment 7 of the liver, and a large cystic lesion with multiple daughter cysts located in the peritoneal cavity near the gastrosplenic region. The latter cyst measured 9.8 x 10.8 x 13.7 cm and was compressing the left kidney and the tail of the pancreas, closely abutting the splenic and left renal vessels (Figures 1A-1D).

**Figure 2:**
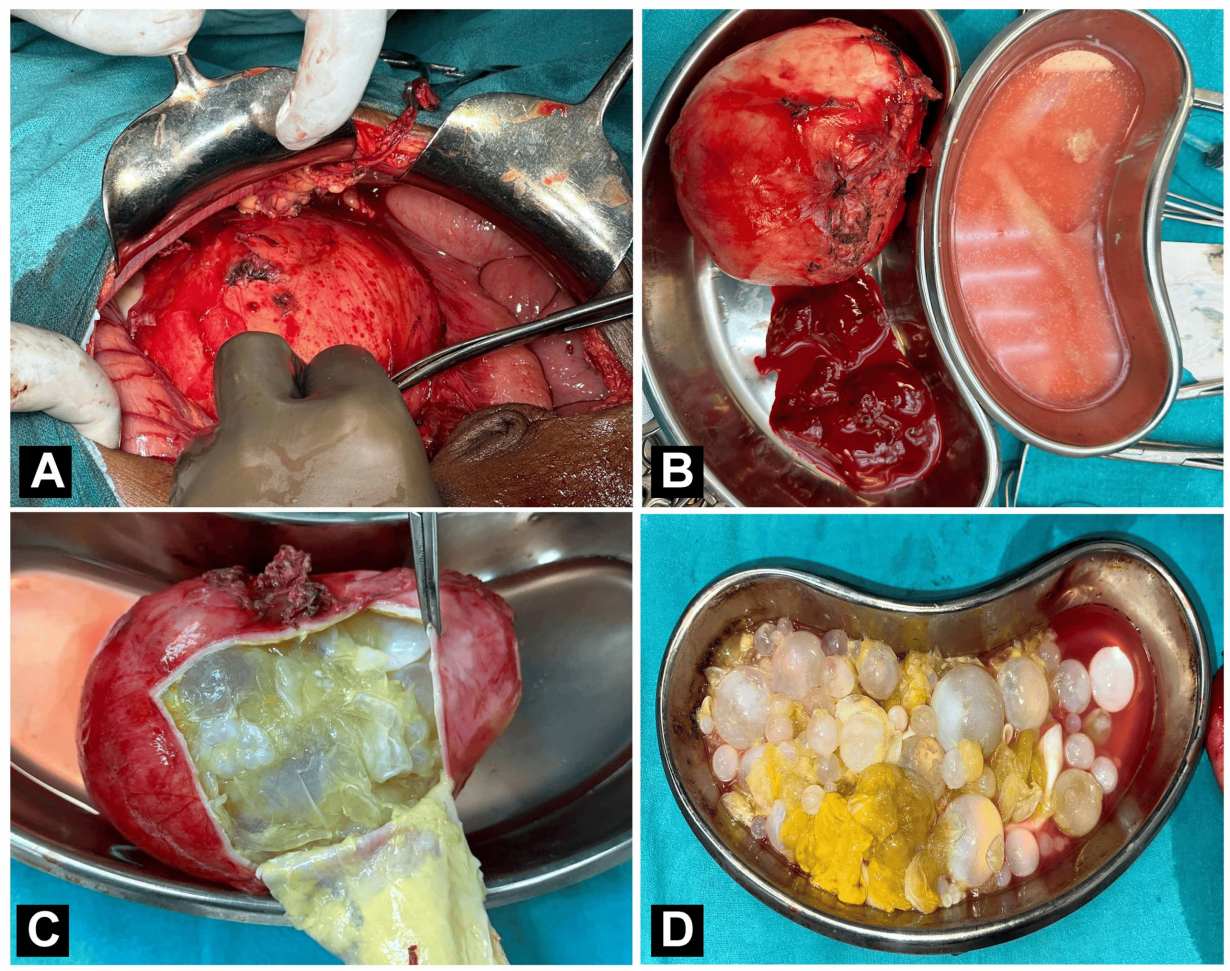
(A) Intraoperative image of the peritoneal hydatid cyst procedure, (B) excised specimen of peritoneal hydatid cyst, and (C, D) peritoneal hydatid cyst showing daughter hydatid cyst.

During the surgical procedure for excision of peritoneal and liver hydatid cysts, a midline vertical incision was made extending from the xiphisternum to 2 cm below the umbilicus. Careful layer-by-layer dissection of the peritoneum followed, ensuring meticulous attention to avoid damage to underlying structures. A large, approximately 13x12x6 cm hydatid cyst was identified within the lesser sac, densely adherent to the omentum, tail of the pancreas, transverse colon, and splenic hilum. The cyst was carefully dissected and mobilized along with its blood supply vessels, which were ligated and cut. Additionally, a 5x5 cm hydatid cyst located in segment 8 of the liver, containing daughter cysts, was excised using a cruciate incision and subjected to pericystectomy (Figures 2A-2D). Post-procedure, a thorough wash with 20% cetrimide-soaked saline was administered to prevent infection. The operation proceeded uneventfully, with no incidences of anaphylaxis reported.

Based on these imaging findings suggestive of hydatid cysts, the patient was initiated on medical management with albendazole (400 mg) for six weeks. However, follow-up ultrasound indicated no reduction in the size of the cysts, necessitating a surgical approach. The patient underwent an uneventful operative procedure for excision of the hydatid cysts in the liver and peritoneal cavity. Postoperatively, she received antibiotics, and analgesics, and was prescribed a prophylactic regimen of albendazole (400 mg PO BD for 21 days per cycle) to prevent recurrence of the parasitic infection.

The patient tolerated the surgical procedure well and was advised on postoperative care, including maintaining local hygiene at the suture site and avoiding heavy lifting. Regular follow-ups were scheduled to monitor her recovery and ensure treatment effectiveness. This case highlights the diagnostic challenges and management complexities associated with hydatid cyst disease, emphasizing the importance of a multidisciplinary approach involving medical therapy and surgical intervention tailored to the individual patient's needs and disease presentation.

## Discussion

Hydatid cyst disease, caused by the larval stage of *E. granulosus*, remains a significant public health concern in endemic regions such as South Asia. This parasitic infection commonly affects the liver and occasionally spreads to other organs, including the peritoneum, lungs, and rarely the brain or bones [1]. The clinical presentation of hydatid cyst disease varies widely, ranging from asymptomatic cysts incidentally detected on imaging to severe complications such as rupture, infection, or compression of adjacent structures [9]. In our case, a 36-year-old South Asian female presented with symptoms consistent with liver and peritoneal involvement of hydatid cysts, including abdominal pain, nausea, and decreased appetite. Radiological imaging, particularly CECT, played a crucial role in confirming the diagnosis by identifying characteristic cystic lesions with daughter cysts, typical of hydatid disease [10].

Treatment strategies for hydatid cyst disease depend on several factors, including cyst size, location, and the presence of complications. Medical therapy with benzimidazoles like albendazole or mebendazole is often initiated to reduce cyst viability and prevent recurrence, although complete resolution of cysts through medical management alone can be challenging [6]. Surgical intervention remains the cornerstone for large or complicated cysts, as in our case, where the inadequate response to medical therapy and the risk of compressive symptoms necessitates surgical excision [11]. Surgical management of hydatid cysts aims to achieve complete cyst removal while minimizing the risk of intraoperative spillage and subsequent anaphylactic reactions. Postoperative care includes the administration of anthelmintics to prevent recurrence, as evidenced by our patient's prophylactic albendazole regimen [12]. Long-term follow-up is essential to monitor for recurrence or complications post-surgery, underscoring the chronic nature of this parasitic infection. The challenges in managing hydatid cyst disease are compounded by diagnostic delays, variability in cyst presentation, and the potential for cyst recurrence even after apparently successful treatment. Multidisciplinary collaboration involving infectious disease specialists, radiologists, and surgeons is crucial for optimal patient outcomes, ensuring timely diagnosis, appropriate treatment selection, and comprehensive postoperative care [13].

## Conclusions

In conclusion, the case of hydatid cyst disease in our South Asian female patient underscores the diagnostic challenges and therapeutic complexities associated with this parasitic infection. The presentation involving both hepatic and peritoneal cysts highlights the varied clinical manifestations and the need for a multidisciplinary approach to management. While medical therapy with albendazole was initially attempted, surgical excision became necessary due to inadequate response and the risk of complications from cyst growth and compression of adjacent structures. Postoperative care, including prophylactic albendazole administration, aimed at preventing recurrence, reflects the chronic nature of hydatid disease and the importance of long-term follow-up. This case emphasizes the significance of timely diagnosis, individualized treatment strategies, and comprehensive postoperative management to optimize outcomes in patients with hydatid cyst disease.
